# A multidimensional model of memory complaints in older individuals and the associated hub regions

**DOI:** 10.3389/fnagi.2023.1324309

**Published:** 2023-12-21

**Authors:** Véronique Paban, A. Mheich, L. Spieser, M. Sacher

**Affiliations:** ^1^Aix-Marseille Université, CNRS, LNC (Laboratoire de Neurosciences Cognitives–UMR 7291), Marseille, France; ^2^CHUV-Centre Hospitalier Universitaire Vaudois, Service des Troubles du Spectre de l’Autisme et Apparentés, Lausanne University Hospital, Lausanne, Switzerland; ^3^University of Toulouse Jean-Jaurès, CNRS, LCLLE (Laboratoire Cognition, Langues, Langage, Ergonomie–UMR 5263), Toulouse, France

**Keywords:** memory complaints, subjective cognitive decline, structural equation modeling, cognitive factors, graph theory

## Abstract

Memory complaints are highly prevalent among middle-aged and older adults, and they are frequently reported in individuals experiencing subjective cognitive decline (SCD). SCD has received increasing attention due to its implications for the early detection of dementia. This study aims to advance our comprehension of individuals with SCD by elucidating potential cognitive/psychologic-contributing factors and characterizing cerebral hubs within the brain network. To identify these potential contributing factors, a structural equation modeling approach was employed to investigate the relationships between various factors, such as metacognitive beliefs, personality, anxiety, depression, self-esteem, and resilience, and memory complaints. Our findings revealed that self-esteem and conscientiousness significantly influenced memory complaints. At the cerebral level, analysis of delta and theta electroencephalographic frequency bands recorded during rest was conducted to identify hub regions using a local centrality metric known as betweenness centrality. Notably, our study demonstrated that certain brain regions undergo changes in their hub roles in response to the pathology of SCD. Specifically, the inferior temporal gyrus and the left orbitofrontal area transition into hubs, while the dorsolateral prefrontal cortex and the middle temporal gyrus lose their hub function in the presence of SCD. This rewiring of the neural network may be interpreted as a compensatory response employed by the brain in response to SCD, wherein functional connectivity is maintained or restored by reallocating resources to other regions.

## Introduction

As individuals reach middle-age and beyond, it is common for them to report memory complaints, with the frequency of these complaints increasing as they age (Jonker et al., 2000). Such complaints may be indicative of early cognitive impairment or dementia (Jessen et al., 2014), as well as depression or anxiety symptoms (Balash et al., 2013). In 2014, an international group of researchers and clinicians, known as the SCD-initiative (SCD-I) (Jessen et al., 2014), termed these complaints subjective cognitive decline (SCD) (many others terms exist for these perceived cognitive problems, including subjective memory complaints, subjective cognitive impairment, subjective memory impairment, functional memory disorder and other terminology). Interestingly, individuals with SCD typically perform within the normal range on neuropsychological tests of memory and attention (Jonker et al., 2000), despite experiencing everyday memory failures such as forgetting names or struggling to find the right word and lack of the ability to concentrate (Snitz et al., 2012). However, the awareness of these memory failures can cause distress, leading to increased stress levels and further lapses in memory (Mizuno et al., 2018). Warwick and Salkovskis (1990) well describe a harmful cycle that individuals with SCD can experience. Because of the lack of impairment on objective tests, cognitive difficulties can only be measured through self-report measures (Rabin et al., 2015). However, these measures vary and can include tasks such as self-ratings of memory or comparing current and past performance. This variation makes it challenging to compare study results and can lead to the interference of other factors. The factors susceptible to interacting with SCD are numerous. One possible factor is the presence of anxiety and depression states. While many older adults are aware of age-related changes in cognition, those with anxiety and/or depression may exhibit hypersensitivity to perceived cognitive failures, potentially leading to an overreporting of complaints. Emotional distress is frequently observed in subjects with SCD (for a comprehensive review, see Knight and Durbin, 2015; Rabin et al., 2015; Desai et al., 2021). Metacognitive beliefs are associated with SCD. Jenkins et al. (2021) propose that individuals with more pronounced SCD tend to exhibit poorer metacognitive function, negative affective symptoms, and higher stress levels. People with SCD commonly experience low self-esteem in memory-related tasks and anxiety regarding their memory in demanding situations (Metternich et al., 2009; Liew, 2020), reinforcing the role of anxiety and highlighting the influence of self-esteem in SCD. The role of personality factors in SCD has been well-documented (for a comprehensive review, see Koller et al., 2019). Notably, neuroticism has been identified as a significant factor in SCD (Comijs et al., 2002). Resilience, denoting the ability to cope in the face of adversity, has been extensively studied in the context of coping with Alzheimer’s disease (AD) pathologies. It has been identified as a factor that may contribute to delaying the onset of cognitive impairment (Arenaza-Urquijo and Vemuri, 2018). In summary, the factors contributing to SCD are numerous and complex, necessitating further evaluation.

SCD has gained attention as a potential early indicator of dementia. Research has shown that SCD is linked to an elevated risk of developing AD and progressive cognitive impairment, as evidenced by various clinical and neuroimaging studies (Jessen et al., 2020; Liew, 2020). Inconsistencies in results were occasionally observed, and these discrepancies may be linked to variations in subject recruitment types. The methods employed in sampling and recruitment play a crucial role in SCD research (Rodríguez-Gómez et al., 2015). Notably, patients sourced from memory clinics often exhibit higher levels of cognitive impairment compared to those from population samples (Kuhn et al., 2019). According to the findings reviewed by Pini and Wennberg (2021), structural imaging studies indicate significant differences in brain atrophy patterns between SCD clinical and community samples. To investigate the key brain regions involved in SCD, biochemical, structural, and functional approaches have been utilized, with several regions such as the medial temporal and occipitoparietal regions being implicated (for review, see Wang et al., 2020). Graph theory is a useful methodology for investigating the human connectome. With this approach, the brain can be conceptualized as a set of nodes interconnected by a set of edges (Bullmore and Bassett, 2011). Graph theory metrics, which are used to assess the functional properties of brain networks at global and nodal levels, provide information on the network’s ability to process information within densely interconnected groups of brain regions (Pereira et al., 2016). These metrics have been used to examine network topology in healthy individuals as well as in those with neuropsychiatric and neurodegenerative conditions such as depression, schizophrenia, and AD (Achard et al., 2006; Kabbara et al., 2017). In SCD, recent investigations have examined brain alterations using graph network properties with functional MRI (Li et al., 2018) and structural MRI data (Fu et al., 2022) during rest. These studies have consistently demonstrated that SCD patients exhibit lower values in local network metrics (e.g., degree, shortest path length, clustering coefficient, local efficiency) compared to controls. However, they also indicate preserved global network properties such as small-worldness, modularity, and transitivity. Notably, only a limited number of studies have investigated graph metrics in SCD and non-SCD populations using magnetoencephalography (MEG) data. López-Sanz et al. (2017, 2018) found decreased clustering in the theta and beta bands, as well as increased transitivity in the alpha band in SCD individuals. Furthermore, at a nodal level, SCD patients exhibited increased activity in the left post-central node in the beta band. Resting-state electroencephalographic (EEG) studies are relatively scarce in this area. EEG stands as a distinctive non-invasive method that, when coupled with advanced signal processing algorithms, is progressively evolving into a promising neuroimaging approach (Michel and Murray, 2012; Hassan and Wendling, 2018). In SCD individuals, using graph theory on EEG data, Lazarou et al. (2020) reported no statistically significant differences at the global level using a wide frequency range (0.3 Hz to 75 Hz), but at the nodal level, SCD individuals showed lower clustering and strength, as well as higher betweenness centrality compared to healthy older individuals. It is important to note that nodal analyses in these studies were only focused on the parietal lobe, highlighting the need for further exploration of network topographical properties in individuals with SCD.

The aim of the current study was to advance our comprehension of individuals with SCD by elucidating potential cognitive/psychologic-contributing factors and characterizing cerebral hubs within the brain network. To identify these potential contributing factors, a structural equation modeling approach was employed to examine the relationships between factors such as metacognitive beliefs, personality, anxiety, depression, self-esteem, resilience, and memory complaints. The composite score derived from this analysis was used to select the participants employed in the brain analysis. This composite score, calculated for each participant, offers the advantage of taking into account the entirety of the factors included in the study and their respective weights. EEG source connectivity was utilized to reconstruct functional brain networks across various frequency bands. Graph theory analyses were applied to assess the topographical organization of brain networks during a resting state. To achieve this objective, we initially measured the similarity between brain networks of individuals with non-SCD and SCD using a newly developed algorithm known as SimiNet (Mheich et al., 2018). This algorithm incorporates the physical positions of nodes in the computation of similarity between two brain graphs. Subsequently, we specifically examined the nodal characteristics of these two networks by analyzing graph centrality measures. Centrality serves as a significant network metric as it reveals the nodes that hold critical positions within the entire network. These nodes, commonly known as network hubs, along with central regions, form a central core that exhibits extensive interconnections. These regions are particularly noteworthy as they are involved in various cognitive functions, making them valuable for investigating network alterations associated with neurological disorders. The exploration of these regions has the potential to provide insights into the underlying mechanisms of such diseases (Achard et al., 2006; Miraglia et al., 2017). In this study, the measure of betweenness centrality (BC) was employed to identify hubs within the brain networks. BC is a local metric that characterizes the centrality of a graph by considering the shortest paths and quantifies the extent to which nodes act as intermediaries between other nodes (Rubinov and Sporns, 2010). Building upon previous research highlighting EEG alterations in the delta and theta frequency bands among individuals with SCD, graph theory analyses were conducted specifically on these two frequency bands. Notably, delta and theta EEG signals have consistently exhibited differences in SCD compared to non-SCD individuals, irrespective of the type of analysis performed, be it spectral power or functional connectivity (Gaubert et al., 2019; Babiloni et al., 2020). Dubois et al. (2016) and Lazarou et al. (2019) have highlighted the scarcity of studies investigating EEG analyses in individuals with SCD compared to non-SCD individuals. Furthermore, discrepant findings have been reported within the existing literature. Some studies have observed an increase in delta and theta power among SCD patients compared to healthy older individuals (Babiloni et al., 2020), while others have demonstrated a decrease in delta power specifically in the frontocentral regions (Gaubert et al., 2019).

The objectives of the present study were to enhance our understanding of individuals with SCD by identifying potential cognitive and psychological factors contributing to their condition and by characterizing hub regions within the brain network. We hypothesized that individuals with SCD, when compared to those without SCD, would exhibit inferior performance in areas such as metacognitive beliefs and personality traits. At the cerebral level, we postulated a reassignment of roles in certain key regions, particularly those within the resting-state networks.

## Materials and methods

### Participants

At first, the study recruited 116 individuals aged 60 or above from the general population through advertisements. The study was carried out in accordance with the Declaration of Helsinki and was approved by the “Comité de Protection des Personnes Sud Méditerranée” (agreement n° 19.09.12.44636-AF), and every subject gave their written informed consent to participate. The participants provided information on their medical history, underwent a neuropsychological test battery, and were interviewed about their functional ability and lifestyle. To be eligible for the study, participants had to be able to live independently (as determined by the Activities of Daily Living scale) and not have any medical, psychiatric, or neurological conditions that could affect brain structure or function, including depression [assessed using the Geriatric Depression Scale (GDS), Yesavage et al., 1982] and anxiety [assessed using the State Trait Anxiety Inventory form Y-A and Y-B (STAI Y), Spielberger et al., 1983]. They also had to perform normally on cognitive tests, not be taking psychoactive medications, and not have any sensory impairments that could interfere with cognitive testing. Participants were excluded from the study if they had a clinical diagnosis of mild cognitive impairment (MCI) or dementia based on self-reported medical diagnosis, self-reported cognitive decline that could be attributed to a psychiatric or neurological disorder, a history of head injury or medical condition, the use of medication (both prescribed and non-prescribed) that could affect cognitive function, or substance abuse. Participants were submitted to a battery of neuropsychological tests and filled out several anonymous self-report questionnaires approximately 2 weeks before EEG experiment.

### Neuropsychological tests

The neuropsychological battery included global cognition assessment with the Mini- Mental State Examination (MMSE) (Folstein et al., 1975), memory evaluation with the RL/RI-16 Test (Grober and Buschke, 1987) and the Boston Naming Test (BNT) (Kaplan et al., 2001), executive abilities with the Stroop Color Word Test (Scarpina and Tagini, 2017), the Digit Span backward (Wechsler, 2018), and the Trail Making Test form A and B (Tombaugh, 2004); fluency measures with the Letter and Category Verbal Fluency test (Benton, 1968), visuo-spatial skills with the Rey-Osterrieth Complex Figure Copy (Berry et al., 1991).

### Questionnaires

The test battery comprised questionnaires to measure (1) cognitive complaints: Cognitive Change Index (Self report) (CCI-S) evaluating one’s memory performance, executive function, and language (Rattanabannakit et al., 2016); Self-assessment of Cognitive Deficits (CDS) (McNair and Kahn, 1983), and Prospective and Retrospective Memory Questionnaire (PRMQ) evaluating one’s Prospective memory, Retrospective memory, self-cued memory, environmentally-cued memory, short-term memory, long-term memory (Guerdoux-Ninot et al., 2019); (2) metacognitive beliefs: Meta-Cognitions Questionnaire 30 (MCQ-30), containing five domains which are Positive Beliefs, Beliefs about Uncontrollability and Danger, Cognitive Confidence, Beliefs related to Superstition, Punishment, and Responsibility, Cognitive Self-Consciousness (Dethier et al., 2017); (3) personality factors: 10-item Big-Five Inventory containing five factors which are Extraversion, Agreeableness, Emotional Stability, Conscientiousness, Openness to Experience (BFI-10) (Courtois et al., 2020); (4) self-esteem: French version of the Rosenberg Self-Esteem Scale (Gana et al., 2005); (5) resilience: Connor-Davidson Resilience Scale (CD-RISC) (Connor and Davidson, 2003).

The participants were divided into two groups, the SCD and non-SCD groups, based on their scores on the CCI-S test. The validated cut-off values for grouping were used, which were established in the Alzheimer’s Disease Neuroimaging Initiative (ADNI) study and can be found on the ADNI website (Risacher et al., 2015).^1^ These cut-offs were based on the first 12 items of the CCI-S that relate to memory concerns (CCI-S memory), and individuals scoring 20 or higher on these items were considered to have memory concerns.

Demographic measures, neuropsychological tests, and questionnaires were analyzed using XLSTAT software. Two-tailed *p*-values were reported, and *t*-tests were used for normally distributed variables, while the Mann-Whitney U test was used for non-normally distributed variables.

### Partial least squares structural equation modeling

To identify potential contributing factors to memory complaints and examine how they relate to each other, an advanced statistical method known as partial least squares structural equation modeling (PLS-SEM) was used. As described previously (Paban et al., 2018), PLS-SEM is an advanced statistical method based on exploratory techniques (Bollen and Lennox, 1991). Unlike other techniques, PLS-SEM does not require making assumptions about data distribution, and it works effectively with small sample sizes and complex models (Hair et al., 2021). The method evaluates the relationship among latent variables or constructs, which are comprised of several items that measure a single concept. By representing the relationships between constructs in a diagram, circles denote constructs, and arrows indicate relationships. The path model is converted into a set of equations that describe a measurement model and a structural model (Haenlein and Kaplan, 2004). PLS-SEM computes a composite score for each participant, which accounts for all the factors included in the model and their respective weights. The validity of the measurement model was assessed using internal consistency, convergent validity, and discriminant validity. Composite reliability of the items was used to calculate internal consistency, and the convergent validity was evaluated based on the average variance extracted (AVE) scores for each construct and the outer loading of each indicator. Discriminant validity was assessed by examining the cross-loading. Regarding the structural model, the quality of the relationships was measured by the R^2^ metric, which measures the level of the explained variance of the composites. Statistical comparisons of path coefficients among the two groups of subjects were performed using multigroup comparison methods offered by the XLSTAT software within the framework of PLS path modeling presented by Goles and Chin (2005). A non-parametric permutation test with 10,000 permutations was used, and statistical significance was set at *p* ≤ 0.05.

### EEG recording and preprocessing

Fifty participants were involved in a resting-state study where EEG data were collected. Among them, 26 participants were classified as belonging to the SCD group, while the remaining 24 participants were categorized as the non-SCD group based on their composite scores provided by the PLS-SEM analysis. Each EEG session consisted of a 5-min period where participants were instructed to close their eyes and relax. Participants were seated in a dimly lit room, were instructed to close their eyes and then to simply relax until they were informed that they could open their eyes. The eyes-closed resting EEG recordings protocol was chosen in order to minimize movement and sensory input effects on electrical brain activity. EEG data were collected using a 64-channel Biosemi ActiveTwo system (Biosemi Instruments, Amsterdam, The Netherlands), following the standard 10–20 system montage. Additionally, two bilateral electro-oculogram electrodes were employed to track horizontal eye movements. Nasion-inion and pre-auricular anatomical measurements were made to locate each individual’s vertex site. Data were digitized at a sampling rate of 1,024 Hz. The impedance of the electrodes was maintained below 20 kOhm.

To preprocess the EEG data, the EEGLAB software was used (Delorme and Makeig, 2004). The recordings were bandpassed (0.5–70 Hz) and undersampled to 256 Hz offline. The recorded EEG signals underwent preprocessing following the PREP pipeline described by Bigdely-Shamlo et al. (2015). Independent component analysis (ICA) was applied to remove artifacts caused by eye blinks, movement, and motion. The independent components (ICs) were classified using the SASICA plugin, which provided detailed information to guide the selection of artifact ICs (Chaumon et al., 2015). The data were then segmented into consecutive epochs of 2 s. The number of rejected ICs varied among participants, ranging from 3 to 10. An average of 100 artifact-free segments, each lasting 2 s, were used for further analysis.

### Graph measures

Graph measures were obtained using several software, including LORETA_KEY (low-resolution electromagnetic tomography) package, SimiNet (Similarity Network), and BRAPH (BRain Analysis using graPH theory). LORETA_KEY enabled the researchers to estimate the intracerebral electrical sources responsible for generating the recorded scalp activity within each of the examined frequency bands (Pascual-Marqui et al., 2002). The exact (e) LORETA method was employed as an inverse solution, providing precise localization of brain activity with minimal errors even in the presence of measured and structured biological noise. For connectivity estimation between regions of interest (ROIs), eLORETA intracortical lagged phase synchronization was selected. These ROIs were defined based on the available Brodmann areas (BAs) in both the left and right hemispheres, resulting in a total of 58 defined ROIs. The decision to rely on Brodmann areas was based on prior literature and the use of software that exclusively employs this method. This approach has been considered appropriate for computing resting-state networks (Babiloni et al., 2016; Paban et al., 2018). Detailed information regarding the eLORETA connectivity algorithm can be found in a previously published work (Pascual-Marqui, 2002). In summary, an EEG source connectivity analysis was conducted by restricting the source space to the gray matter, encompassing 6,239 voxels with a spatial resolution of 5 mm, as defined by the digitized MNI152 template. We used a template source-space approach rather than a subject-specific one, guided by prior findings indicating that for healthy subjects, co-registration with the template resulted in notably consistent connectivity and network estimates compared to native MRI data (Douw et al., 2018). For each participant, undirected weighted adjacency matrices were calculated within two frequency bands: delta (1–4 Hz) and theta (4–8 Hz).

SimiNet algorithm was used to compute the similarity scores between brain networks (Mheich et al., 2018). The algorithm incorporates both nodes and edges in its computation of the similarity index. In terms of nodes, the algorithm follows four key steps: (i) identification of common nodes shared between the two compared graphs, (ii) substitution of nodes with a substitution cost equivalent to the distance between the replaced nodes, (iii) insertion of new nodes with an insertion cost equal to a constant value, and (iv) deletion of nodes with a suppression cost equal to the insertion cost. The second step involves calculating the distance between edges. This entails summing the differences in weight between corresponding edges in the two compared graphs. The algorithm generates a normalized similarity index, where 0 signifies no similarity and 1 represents two identical networks with the same properties and topology. Analyses were done for the delta and theta frequency bands.

The computation of graph measures in this study was performed using the BRAPH software (Mijalkov et al., 2017). In this analysis, the nodes of the graph were defined as the voxel centroids corresponding to the 58 regions of interest (ROIs). The edges of the graph were established based on the lagged phase synchronization values between the nodes. Graph topological properties were assessed on the fully weighted undirected network for the delta and theta frequency bands. In this study, the assessment of betweenness centrality (BC) was conducted throughout the entire brain. BC is a local metric that captures the functional relationships of a specific node within the entire brain’s connectivity matrix (Rubinov and Sporns, 2010). More specifically, BC quantifies the fraction of all shortest paths in the graph that include a particular node. Nodes with higher BC values are involved in a greater number of shortest paths, indicating their importance in facilitating efficient information flow within the network. Hub regions were defined as nodes with BC values at least 1.5 standard deviations higher than the mean. The resulting lists of ROIs were used to construct a Venn diagram, a graphical representation that elucidates the relationships between different sets or groups of items, facilitating the comprehension of how two or more sets interrelate, thereby simplifying the identification of shared properties and distinctions. The Venn diagram yielded quantitative information, including the number of ROIs in both the non-SCD and SCD groups, as well as the size of the intersection. We categorized ROIs into three types: (1) ROIs found in non-SCD, representing hubs observed in healthy older individuals; (2) ROIs highlighted in SCD, representing hubs associated with memory complaints; and (3) overlapped ROIs, indicating common hubs in older subjects, whether or not they had memory complaints.

## Results

### Neuropsychological analysis

From the original sample, 95 participants, between 60 and 86 years (mean age of 69.89 ± 0.95 years; 73 woman), were enrolled in the study. All of them completed the battery of neuropsychological tests and questionnaires. Education ranged from 8 years of schooling to a PhD degree. The main demographical and neuropsychological characteristics of all subjects (*N* = 95) are summarized in Table 1. There were no differences between SCD and non-SCD participants with regard to age, gender, and years of education. Both groups exhibited normal results on neuropsychological evaluations, as SCD individuals typically perform within the expected range on such tests. However, it is noteworthy that the SCD group demonstrated significantly lower performance compared to the non-SCD group in the free recall portion of the RL/RI test and the Letter verbal fluency test (*p* ≤ 0.03).

**TABLE 1:** Group means and standard deviation (SD) of demographical and neuropsychological characteristics of non-SCD and SCD groups (*N* = 95).

	Non-SCD	SCD	*p*
	*n* = 45 Men/Women: 11/34 Mean (S.D)	*n* = 50 Men/Women: 11/39 Mean (S.D)	
Age	69.75 ± 5.86	71.02 ± 7.33	0.10
Education (years)	14.11 ± 3.14	14.56 ± 3.04	0.48
CCI-S memory	16.31 ± 3.41	32.34 ± 5.03	<0.0001
**Neuropsychological tests**			
MMSE	29.51 ± 0.59	29.24 ± 1.12	0.14
**RL/RI-16 test**			
Free recall	11.27 ± 1.68	10.09 ± 2.20	0.004
Recognition	15.87 ± 0.40	15.74 ± 0.83	0.35
Rey-Osterrieth Complex Figure recall	20.42 ± 5.83	20.26 ± 7.22	0.91
**Verbal Fluency test**			
Category	35.56 ± 7.75	32.78 ± 8.16	0.09
Letter	24.93 ± 7.38	21.88 ± 6.18	0.03
**Trail Making Test**			
Form A	41.33 ± 3.66	36.91 ± 1.83	0.36
Form B	80.05 ± 6.33	77.32 ± 5.42	0.66
Stroop	54.38 ± 8.18	47.86 ± 9.70	0.29
Digit Span backward	4.36 ± 0.93	4.12 ± 0.82	0.19
Boston Naming Test	54.49 ± 4.64	56.02 ± 3.99	0.09

### Partial least squares structural equation modeling analysis

The measurement model was elaborated using data from all 95 participants. Initially, a model was tested with all variables included, but variables that did not significantly contribute were eliminated, resulting in a more restrictive model. Specifically, six of height indicators in the ‘Neuropsychological tests’ construct were removed, leaving only scores from the free recall of the RL/RI-16 Test and the Letter and Category Verbal Fluency test. Agreeableness, Extraversion, and Openness to Experience were also eliminated from the ‘Personality factors’ construct.

Table 2 presents the results of the reliability and validity assessment of the second model, which includes five reflective constructs: memory complaints, neuropsychological tests, emotional distress (depression and anxiety), metacognitive beliefs, and personality factors. The internal consistency measures, as indicated by the composite reliability, were found to be between 0.71 and 0.96, surpassing the recommended threshold value of 0.70. The convergent validity was satisfactory, with an AVE value above 0.5 for all four constructs. Each item’s factor loading was significant (*p* < 0.05, data not shown), and all but one was above 0.6.

**TABLE 2:** Assessment of the measurement model: internal consistency (composite reliability), convergent validity [loading and average variance extracted (AVE)], and discriminant validity (cross-loading).

Composite reliability			Outer loadings and cross loadings				
Latent variables	Indicators	Memory	Neuropsy.	Emotional	Metacog.	Perso.	AVE
Memory complaints	0.96						0.77
CDS		**0.88**	-0.37	0.55	0.57	-0.51	
CCI-S Memory		**0.81**	-0.37	0.42	0.43	-0.44	
CCI-S Executive		**0.74**	-0.27	0.53	0.43	-0.53	
CCI-S Langage		**0.74**	-0.45	0.46	0.45	-0.5	
PMRQ Prospective memory		**0.93**	-0.28	0.39	0.55	-0.39	
PMRQ Retrospective memory		**0.91**	-0.29	0.34	0.49	-0.42	
PMRQ short-term memory		**0.93**	-0.28	0.37	0.53	-0.42	
QMPR long-term memory		**0.94**	-0.31	0.35	0.54	-0.39	
PMRQ self-cued memory		**0.93**	-0.28	0.38	0.53	-0.38	
PMRQ environmentally-cued memory		**0.91**	-0.31	0.33	0.53	-0.42	
Neuropsychological tests	0.71						0.56
Free-Recall of the RL/RI-16 Test		-0.38	**0.85**	-0.26	-0.31	0.16	
Category Verbal Fluency test		-0.21	**0.75**	-0.13	-0.16	0.06	
Letter a Verbal Fluency test		-0.18	**0.62**	-0.29	-0.12	0.24	
Emotional distress	0.81						
GDS		0.46	-0.29	**0.82**	0.4	-0.61	0.72
STAI Y-A		0.29	-0.3	**0.84**	0.38	-0.6	
STAI Y-B		0.42	-0.18	**0.88**	0.56	-0.7	
Metacognitive beliefs	0.76						0.5
MCQ-30 Positive Beliefs		0.21	-0.42	0.43	**0.63**	-0.23	
MCQ-30 Beliefs about Uncontrollability and Danger		0.33	-0.1	0.46	**0.75**	-0.24	
MCQ-30 Cognitive Confidence		0.64	-0.19	0.35	**0.77**	-0.36	
MCQ-30 Punishment		0.32	-0.28	0.41	**0.76**	-0.23	
MCQ-30 Cognitive Self-Consciousness		0.15	-0.08	0.24	**0.5**	-0.08	
Personality factors	0.72						0.55
SES		-0.42	0.14	-0.71	-0.39	**0.86**	
CD-RISC		-0.27	0.09	-0.53	-0.21	**0.71**	
BFI-10 Conscientiousness		-0.35	0.02	-0.25	-0.04	**0.64**	
BFI-10 Emotional stability		-0.34	0.18	-0.59	-0.33	**0.72**	

CDS, self-assessment of cognitive deficits; CCI-S, cognitive change index self report; PMRQ, Prospective and Retrospective Memory Questionnaire; MCQ-30, Meta-Cognitions Questionnaire 30; BFI-10, 10-item Big-Five Inventory; GDS, Geriatric Depression Scale; STAI Y-A, Y-B: State Trait Anxiety Inventory form Y-A and Y-B; SES, Self-Esteem Scale; CD-RISC, Connor-Davidson Resilience Scale.

**FIGURE 1:**
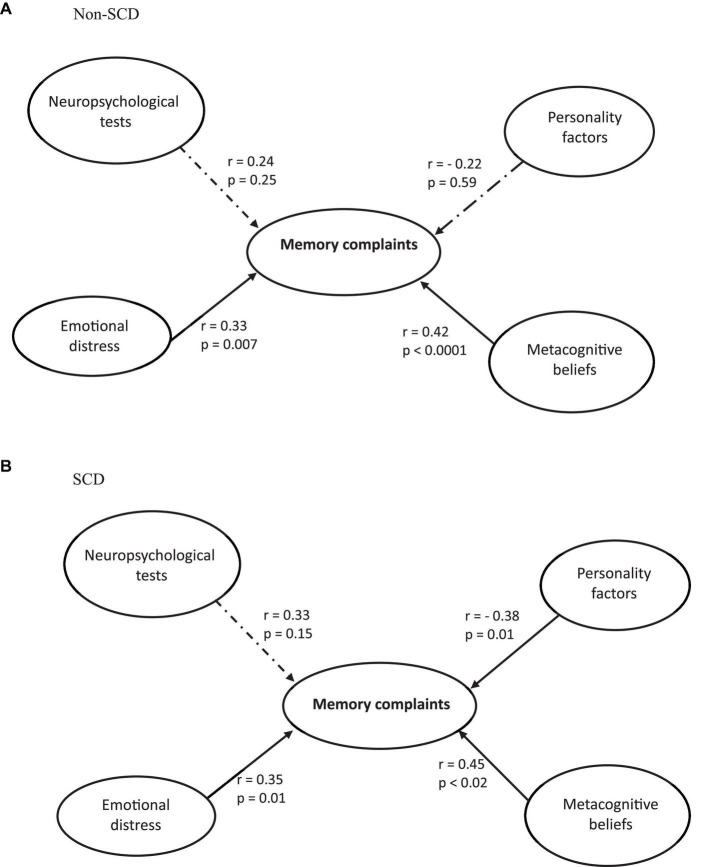
Structural model of the direct effects of memory complaints on neuropsychological tests, emotional distress, metacognitive beliefs, and personality factors in non-SCD **(A)** and SCD **(B)** participants.

After establishing the validity of the measurement model, the structural model was evaluated for each of the two groups (SCD and non-SCD) separately, and the results are presented in Figure 1. In the non-SCD group (Figure 1A), the model was found to be statistically significant (*F* = 6.64, *p* < 0.0001), with an R^2^ value of 0.40. Memory complaints were found to have a significant link with Emotional distress and Metacognitive beliefs (*r* ≥ 0.33; *p* ≤ 0.007). Thus, individuals with higher levels of emotional distress are associated with increased reports of memory complaints. Similarly, higher levels of metacognitive beliefs are associated with higher levels of memory complaints. The relationships between Neuropsychological tests and Personality factors and Memory complaints were not statistically significant (*p* ≥ 0.25).

In the SCD group (Figure 1B), the model was statistically significant (*F* = 5.97, *p* < 0.001), with an explained variance of *R*^2^ = 0.35. Memory complaints had a significant positive correlation with both Emotional Distress and Metacognitive Beliefs (*r* ≥ 0.35; *p* ≤ 0.02), suggesting that the greater the emotional distress or the more substantial the metacognitive beliefs, the higher the level of memory complaints. Memory complaints demonstrated a significant negative correlation with Personality Factors (*r* = −0.38; *p* = 0.01), signifying that the higher the scores on personality factors, the lower the levels of memory complaints No significant effect was yielded for Neuropsychological tests (*p* = 0.15).

The results of the permutation test revealed a significant distinction between the two groups of subjects for the “memory complaints – personality factors” path (*p* = 0.04), indicating that among all the factors analyzed, only the relationship between memory complaints and personality was significantly different between the non-SCD and SCD groups. In other words, personality factors have an influence only in the SCD group. Specifically, the Rosenberg Self-Esteem Scale and the Conscientiousness dimension of the BFI-10 were significantly different between the non-SCD and SCD groups (*p* < 0.01), suggesting that the lower the scores on these two factors, the higher the levels of memory complaints.

**FIGURE 2:**
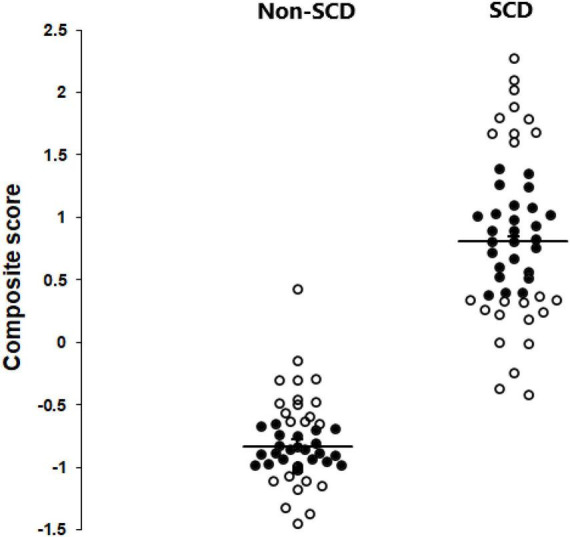
Scattergrams depicting the distribution of all 95 subjects, both with and without SCD, using the PLS-SEM composite score. The 50 subjects (24 non-SCD and 26 SCD) chosen for subsequent EEG and graph analysis are highlighted in black circles.

Partial least squares structural equation modeling allows for the development of a composite score that takes into account all the studied factors and their respective weights. Figure 2 depict the distribution of all 95 subjects, both with and without SCD, using this composite score. The unit is arbitrary. The higher the score, the higher the level of memory complaints. Based on this, only a limited number of 50 non-SCD and SCD subjects were selected for subsequent EEG and graph analysis (highlighted in black circles). The underlying concept was to concentrate on subjects in the middle of the distribution. Our primary interest was indeed to understand the behavior and characteristics of typical or average cases within a population. By excluding extremes, we can ensure that our analysis focuses on the central tendencies of the data, which may be more representative of the majority of the population.

### Graph analysis

**FIGURE 3:**
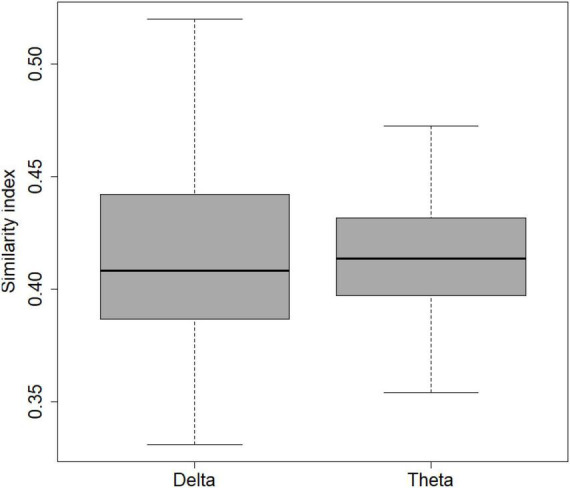
Boxplots showing similarity index of SCD vs. non-SCD in delta and theta bands.

The demographic and neuropsychological characteristics of the 50 subjects used in EEG and graph analysis are shown in Table 3. Figure 3 presents the results derived from applying SimiNet to compare the brain networks of individuals with SCD and the averaged healthy control network. The similarity index for both delta (sim = 0.41 ± 0.01) and theta (sim = 0.42 ± 0.006) bands was below 0.50 (Mheich et al., 2018), indicating that the structure of the brain networks was different in older individuals with SCD compared to those without SCD.

**TABLE 3:** Group means and standard deviation (SD) of demographical and neuropsychological characteristics of the 50 subjects selected for EEG and graph analysis.

	Non-SCD	SCD	*p*
	*n* = 24 Men/Women: 10/14 Mean (S.D)	*n* = 26 Men/Women: 9/17 Mean (S.D)	
Age	68.92 ± 4.76	71.46 ± 8.06	0.09
Education (years)	13.62 ± 3.16	14.46 ± 3.08	0.39
Composite score	−0.61 ± 0.06	1.15 ± 0.16	<0.0001
**Neuropsychological tests**			
MMSE	29.62 ± 0.64	29.31 ± 1.15	0.24
**RL/RI-16 test**			
Free recall	12.34 ± 1.32	10.09 ± 2.20	0.05
Recognition	15.83 ± 0.38	15.57 ± 1.11	0.28
Rey-Osterrieth Complex Figure recall	20.11 ± 7.95	19.46 ± 7.85	0.77
**Verbal Fluency test**			
Category	35.96 ± 8.04	31.35 ± 8.54	0.08
Letter	23.95 ± 6.98	21.57 ± 6.21	0.25
**Trail Making Test**			
Form A	38.75 ± 2.24	40.69 ± 1.77	0.41
Form B	79.14 ± 5.83	78.82 ± 6.17	0.47
Stroop	41.51 ± 8.14	46.46 ± 7.71	0.56
Digit Span backward	4.08 ± 0.71	4.11 ± 0.76	0.87
Boston Naming Test	55.79 ± 3.78	56.38 ± 3.73	0.58

**FIGURE 4:**
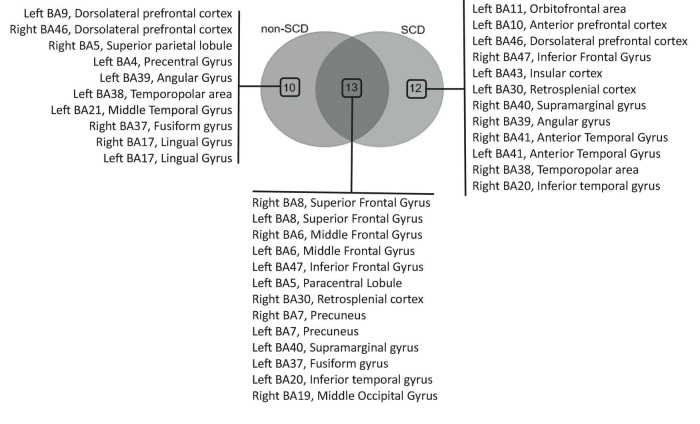
Venn diagram analysis of hub regions of interest (ROIs) in the theta frequency band. The diagram illustrates the overlapping hubs among individuals without (non-SCD) and with SCD, as well as the hubs exclusively identified in each group.

In the delta frequency band, a total of 23 regions of interest (ROIs) were identified as hubs in non-SCD, while 25 hubs were found in SCD (Supplementary Figure 1). The Venn diagram revealed that 13 hubs were shared between the two groups, while 10 ROIs were exclusively identified in the non-SCD group and 12 in the SCD group only (Figure 4). These overlapping hubs were distributed across all lobes of the brain. Specifically, within the frontal lobe, three main hubs were uncovered: the superior (BA 8), middle (BA 6), and left inferior (BA 47) frontal gyri. In the parietal lobe, hubs included the retrosplenial cortex (BA 30), the precuneus (BA 7), and left supramarginal gyrus (BA 40) notably. Within the temporal lobe, the fusiform gyrus (BA 37) and the left inferior temporal (BA 20) were identified as hubs. Only one ROI in the occipital lobe, specifically the right middle occipital gyrus (BA 19), was defined as a hub.

Other ROIs, such as the dorsolateral prefrontal cortex (BA 9/BA 46), the left angular gyrus (BA 39), the left middle temporal gyrus (BA 21), and the left temporopolar area (BA 38) notably, were impacted in their role as hubs due to the presence of memory complaints. In fact, these ROIs, previously identified as hubs in individuals without SCD, ceased to function as hubs in individuals with SCD. In contrast, certain other ROIs emerged as hubs specifically in individuals with SCD. These hubs included the left orbitofrontal area (BA 11), the left dorsolateral prefrontal cortex (BA 46), and the right inferior frontal gyrus (BA 47) notably. In the parietal lobe, the right supramarginal gyrus (BA 40) emerged as hub. Within the temporal lobe, the hubs identified were the anterior temporal gyrus (BA 41) and the right inferior temporal gyrus (BA 20).

**FIGURE 5:**
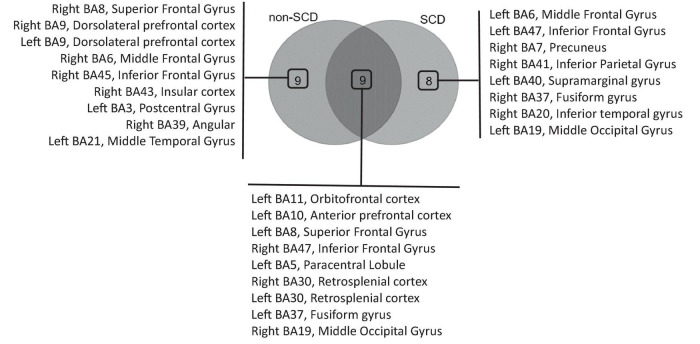
Venn diagram analysis of hub regions of interest (ROIs) in the delta frequency band. The diagram illustrates the overlapping hubs among individuals without (non-SCD) and with SCD, as well as the hubs exclusively identified in each group.

Within the Theta band, 18 ROIs were identified as hubs in non-SCD, while 17 hubs were found in SCD (Supplementary Figure 2). A Venn diagram demonstrated that 9 hubs were shared between the two groups, while 9 ROIs were exclusive to the non-SCD group, and 8 were exclusive to the SCD group (Figure 5). These overlapped hubs were distributed across all lobes of the brain. Specifically, in the frontal lobe, four hubs were identified: the left orbitofrontal cortex (BA 11), the left anterior prefrontal cortex (BA 10), and left superior (BA 8) and the right inferior (BA 47) frontal gyri. In the parietal lobe, the right and left retrosplenial cortex (BA 30) were highlighted as hubs. In the temporal lobe, the left fusiform gyrus (BA 37) emerged as a hub. In the occipital lobe, only one ROI, the right middle occipital gyrus (BA 19), was defined as a hub.

Similar to the findings in the delta band, the presence of memory complaints affected the role of other ROIs. These included the left and right dorsolateral prefrontal cortex (BA 9), right superior (BA 8), right middle (BA 6), and right inferior (BA 45) frontal gyri, as well as the right insular cortex (BA 43) and the right angular gyrus (BA 39) notably. These ROIs, which were previously identified as hubs in non-SCD, lost their hub status in SCD.

In contrast, a distinct set of ROIs emerged as hubs specifically in individuals with SCD. Notably, these hubs included the left middle (BA 6) and left inferior (BA 47) frontal gyri. In the parietal lobe, the right precuneus (BA 7) and the left supramarginal gyrus (BA 40) emerged as hubs. Within the temporal lobe, the identified hubs were the right fusiform gyrus (BA 37) and the right inferior temporal gyrus (BA 20).

## Discussion

The present study aimed to examine the potential cognitive/psychologic-contributing factors to SCD and the neural correlates in terms of hub regions. The findings revealed that personality factors, such as self-esteem and conscientiousness, played a significant role. Network topography differed between the SCD and non-SCD groups at low frequencies bands, namely delta and theta. Another unique aspect of this study is demonstrating that specific brain regions undergo changes in their hub roles as a consequence of the pathology. Some regions become hubs, whereas others lose their hub function following SCD.

To identify potential cognitive and psychological factors associated with memory complaints, we employed structural equation modeling, which encompassed neuropsychological assessments, emotional distress (depression and anxiety), metacognitive beliefs, and personality factors. No significant correlation was found between the neuropsychological evaluation and memory complaints in both individuals with SCD and those without SCD. These findings are consistent with previous research in the literature, which has consistently reported normal performance on standardized cognitive tests among individuals with SCD (for review see Jessen et al., 2020). The reviews conducted by Reid and Maclullich (2006) and Burmester et al. (2016) have indicated that memory complaints are only weakly associated with current cognitive impairment but show a stronger correlation with the risk of future cognitive decline. These findings suggest that individuals who report memory complaints may be at a higher risk for developing cognitive decline in the future. Furthermore, the study revealed a positive correlation between memory complaints and emotional distress in both individuals with SCD and those without SCD. This implies that higher levels of emotional distress are associated with increased reports of memory complaints. It is worth noting that the literature extensively documents the relationship between aging and conditions such as depression and anxiety (for review see, e.g., Knight and Durbin, 2015; Desai et al., 2021). Anxiety and depression are highly prevalent in later life and often co-occur as comorbid disorders, leading to various adverse consequences for individuals (Vink et al., 2008). In the case of participants with SCD, Balash et al. (2013) found a significant association between SCD and affective symptomatology, as measured by scores on the Geriatric Depression Scale (GDS) and State-Trait Anxiety Inventory (STAI). It is important to note that, in the current study, the anxiety and depression scores for all participants were within the “normal” range, indicating that the emotional distress experienced by individuals was sub-clinical. Despite this, there was a significant correlation between emotional distress and memory complaints in both SCD and non-SCD participants. These findings suggest that even when the measures of emotional distress are not elevated, they can still have a significant impact on memory complaints. This highlights the high sensitivity of the Partial Least Squares Structural Equation Modeling (PLS-SEM) approach used in the analysis, as it can detect meaningful effects even with relatively low variability in the measures. It is noteworthy that memory complaints showed a positive correlation with metacognitive beliefs in both individuals with SCD and those without SCD. This indicates that higher levels of memory complaints are associated with higher levels of unhelpful metacognitive beliefs. In the context of traditional cognitive models, metacognitive beliefs refer to individuals’ knowledge and beliefs about the appraisal, monitoring, and control of their own thoughts (Flavell, 1979). According to Palmier-Claus et al. (2011), metacognitive beliefs can be burdensome and may sometimes be irrational or unreasonable, placing undue weight on individuals’ own thoughts. Problematic metacognitive beliefs have been observed in various psychopathological conditions, including anxiety- and mood-related disorders, as well as psychotic disorders (Cotter et al., 2017). The findings of the current study are in line with previous research indicating that both aging individuals with SCD and those without SCD tend to have more negative beliefs about their memory (Hertzog and Dunlosky, 2011; Gautier et al., 2022). These negative beliefs may contribute to their pessimistic evaluation of everyday memory functioning and a decrease in self-efficacy beliefs regarding their memory performance (Zanardo et al., 2006). At the cerebral level, Zhuang et al. (2022) proposed that variations in cortical thickness in temporal, parietal, and medial regions may be associated with metacognitive abilities in older adults without cognitive impairment. Our study revealed a noteworthy finding that demonstrates a significant relationship between memory complaints and personality factors in individuals with SCD. Within the construct of personality factors, two variables showed a significant impact: self-esteem and conscientiousness. These results highlight the influence of self-perception and conscientiousness on memory complaints in individuals with SCD. Self-esteem comprises an individual’s self-conception, encompassing a combination of thoughts and emotions pertaining to their own identity (AlHarbi, 2022). Our findings regarding the association between self-esteem and memory complaints in individuals with SCD align with previous studies conducted by Pearman and Storandt (2004) and dos Santos et al. (2012), and more recently Kim et al. (2021). These studies suggest that individuals’ perception of their memory abilities may be influenced by their self-esteem, or conversely, that self-esteem may be influenced by their perceived memory abilities. The direction of causality in this relationship remains unclear and requires further investigation. Among the five dimensions of personality traits, neuroticism has been extensively studied and consistently shown to be correlated with memory complaints (for review see Koller et al., 2019). In our study, high neuroticism was associated with the memory complaints construct, and this relationship was observed in both the SCD and non-SCD groups. However, only conscientiousness showed a significant correlation with memory complaints specifically in the SCD group, which supports previous findings indicating a negative association between conscientiousness and SCD (Koller et al., 2019). In other words, individuals with higher levels of conscientiousness may potentially experience reduced levels of subjective cognitive decline. Conscientiousness is a personality trait characterized by qualities such as persistence, self-discipline, organization, achievement orientation, and a deliberate approach to tasks and responsibilities (Carver and Connor-Smith, 2010). Growing evidence suggests that conscientious people tend to lead longer and healthier lives [as reviewed by Aschwanden et al. (2021)]. They tend to engage in behaviors that support cognitive health, such as staying physically active, participating in mentally stimulating activities, and maintaining healthy habits. Additionally, conscientiousness may contribute to better coping mechanisms and adaptation when confronted with cognitive challenges. Individuals with high levels of conscientiousness are more inclined to seek support, employ effective problem-solving techniques, and engage in behaviors that enhance overall wellbeing (Flynn and Smith, 2007). It could be speculated that conscientiousness potentially lessens the impact of subjective cognitive decline due to the health behaviors and social environmental factors associated with being conscientious.

The topographical organization of brain networks in individuals with and without SCD was analyzed at both the global and nodal levels and at two frequency bands, delta and theta. The subjects utilized for EEG and graph analysis were chosen based on the composite score derived from the PLS-SEM analysis. Therefore, the identification of individuals with SCD is based not solely on a single criterion, which is the score obtained from the CCI-S memory questionnaire, but rather on a broader set of factors, each considered along with its respective influence weight. The results indicated that, at a global level, as assessed by the similarity index, the brain network of individuals with SCD differed from that of individuals without SCD in both frequency bands. The changes in subjective memory, along with personality factors such as self-esteem and consciousness, were associated with modifications in the overall organization of the network in terms of node, edge, and/or spatial features. In order to go further into our investigation, we sought to identify hub regions using a local centrality metric known as betweenness centrality. The originality of this study is its ability to demonstrate that certain brain regions undergo a change in their hub role due to the pathology. Some regions transition into hubs, while others lose their hub function following SCD. One may speculate that such changes in hub roles could underly a redistribution of functional responsibilities within the brain. In other words, regions that lose their hub function may experience a decline in their ability to integrate and coordinate information across the brain network. Conversely, the regions transitioning into hubs may take on new responsibilities for information integration and become more critical for overall brain functioning. To the best of our knowledge, no such analysis had been conducted before. Among regions that experienced a decline in their hub function, both the left and right dorsolateral prefrontal cortex were found to be active in the delta and theta frequency bands. The dorsolateral prefrontal cortex plays a role in a wide array of cognitive processes, ranging from goal-directed thinking and executive functions to mind-wandering and the psychedelic experience in healthy individuals (Zamani et al., 2022). It is a crucial component of the default-mode network (DMN), along with the temporal pole and the middle temporal gyrus. After experiencing memory complaints, these regions were no longer classified as hubs. Considering their diverse functions within the brain, one could speculate that this change in status may explain the alterations observed in SCD, particularly the issue with conscientiousness mentioned above. Existing literature data have indicated that the SCD group exhibited changes in functional connectivity within the DMN (Lee et al., 2023). Conversely, the right inferior temporal gyrus was the sole region detected in both the delta and theta frequency bands that became a hub in SCD. The inferior temporal gyrus participates in the ventral streams of visual processing and has a crucial role in cognition due to its connections with other cortical areas (Lin et al., 2020). There were other significant brain regions identified as hubs only in the delta or theta frequency bands following memory complaints. For instance, the left orbitofrontal area, which is implicated in emotion and executive function, was highlighted (Rudebeck and Rich, 2018). Additionally, the precuneus, which is recognized for its involvement in a broad range of tightly integrated tasks such as visuo-spatial imagery, episodic memory retrieval, and self-related mental operations including self-consciousness and self-processing during periods of rest, was also implicated (Cavanna and Trimble, 2006). Alterations in parietal regions have been reported by Lazarou et al. (2020). Interestingly, many of the hubs identified in this experiment have previously been reported by Lemaitre et al. (2012) as regions that display age-related decreases in volume, thickness, and surface area. Specifically, the left and right inferior, middle, and superior frontal gyri, along with the precuneus and the inferior parietal gyrus, were identified. Given that the participants in the current study had an average age of 69.89 ± 0.95, it is not surprising that such regions were revealed. Overall, the observed changes in certain brain regions, particularly regarding their hub role, suggest the possibility of neural network rewiring. This rewiring could be seen as a response employed by the brain in the face of SCD, wherein the brain attempts to preserve or reinstate functional connectivity by redistributing its resources to other regions. Identifying these hubs could hold significance in optimizing the effectiveness of therapeutic interventions, such as neurofeedback or transcranial stimulation, which target specific brain regions and their activity and have been used with success in SCD (Chen et al., 2020; Pei et al., 2020).

### Study limitations

In delineating the factors within the PLS-SEM model, our study aimed to comprehensively capture the multifactorial nature of SCD, guided by existing literature. However, it is crucial to acknowledge the inherent limitations in our approach. The selection of factors, though extensive, does not claim exhaustiveness, recognizing the dynamic nature of SCD. Moreover, reliance on self-reports introduces subjectivity, influenced by unexplored moderating factors like cultures and situations.

In the EEG analysis, ROIs were defined using available Brodmann areas due to unavailability of native MRI data, potentially impacting spatial precision. While the template-based method for EEG/MEG connectivity has shown reliability (Hassan et al., 2014), the absence of native MRI for source connectivity limits the methodological ideal. Our focus on delta and theta frequency bands in EEG analysis, while revealing consistent differences in individuals with SCD, emphasizes a potential limitation as other frequency bands, notably the alpha band, remain unexplored. A comprehensive understanding of SCD’s neural correlates necessitates future investigations into a broader spectrum of EEG frequency bands.

## Data availability statement

The raw data supporting the conclusions of this article will be made available by the authors, without undue reservation.

## Ethics statement

The studies involving humans were approved by the Comité de Protection des Personnes Sud Méditerranée (agreement n^°^ 19.09.12.44636-AF). The studies were conducted in accordance with the local legislation and institutional requirements. The participants provided their written informed consent to participate in this study.

## Author contributions

VP designed the study, acquired data, analyzed and interpreted data, wrote the manuscript, and obtained funding. AM performed SimiNet analysis. LS helped for EEG preprocessing. MS acquired data.

## Footnotes

1
http://www.adni-info.org

## Supplementary material

Supplementary Figure 1 Betweenness centrality (BC) scores of the 58 regions of interest (ROIs) in non-SCD and SCD group within the delta frequency band. The dotted line indicates the cut-off threshold. ROIs with BC scores above this cut-off were considered as hubs.

Supplementary Figure 2 Betweenness centrality (BC) scores of the 58 regions of interest (ROIs) in non-SCD and SCD group within the theta frequency band. The dotted line indicates the cut-off threshold. ROIs with BC scores above this cut-off were considered as hubs.
