# Supplementary figures and images for: A multidimensional model of memory complaints in older individuals and the associated hub regions
Source: Front Aging Neurosci. 2023 Dec 21;15:1324309. doi: 10.3389/fnagi.2023.1324309 (PMC10771290; doi:10.3389/fnagi.2023.1324309)

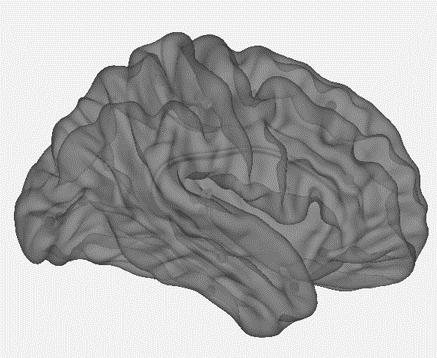

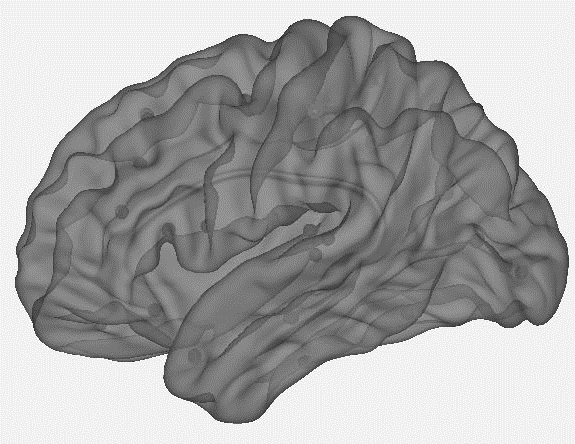


**Right brain**

**Left brain**


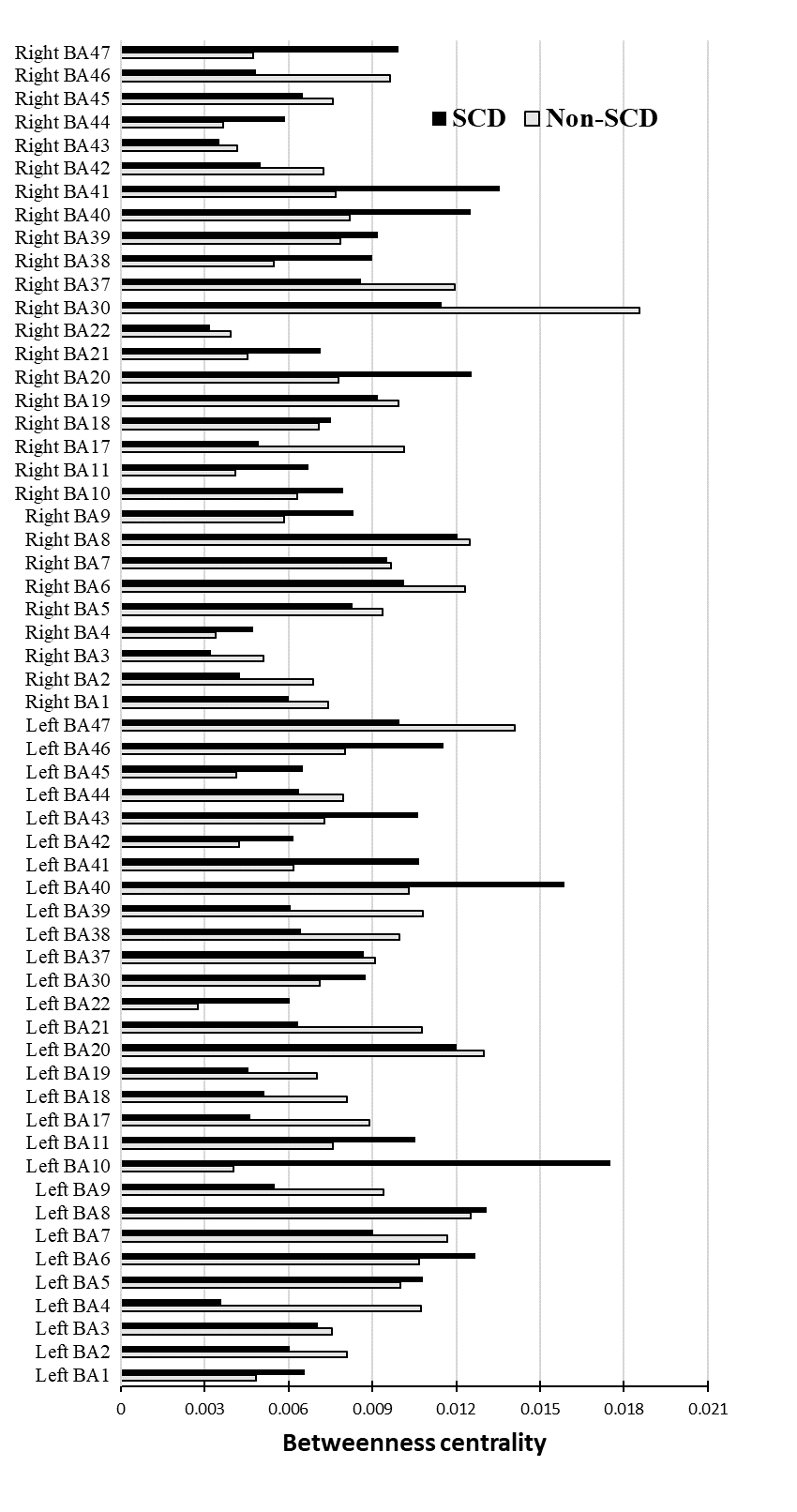

Supplement: Supplementary Figure 1 — Betweenness centrality (BC) scores of the 58 regions of interest (ROIs) in non-SCD and SCD group within the delta frequency band. The dotted line indicates the cut-off threshold. ROIs with BC scores above this cut-off were considered as hubs. [file Data_Sheet_1.docx]

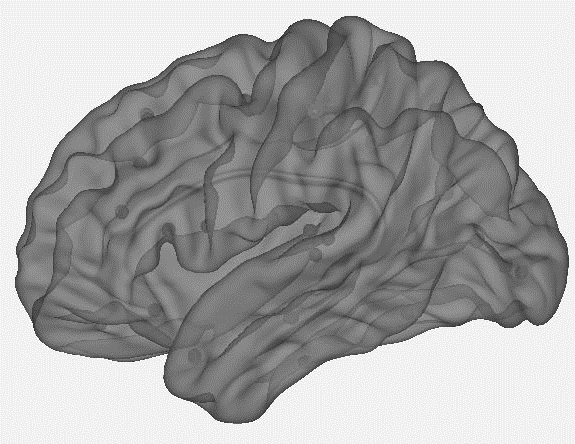

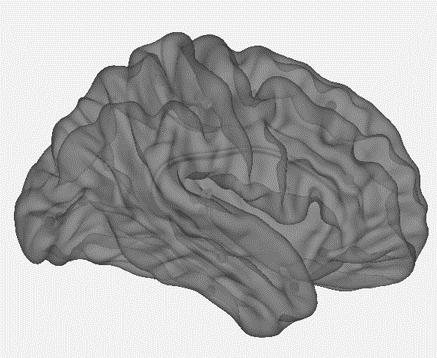


**Left brain**

**Right brain**


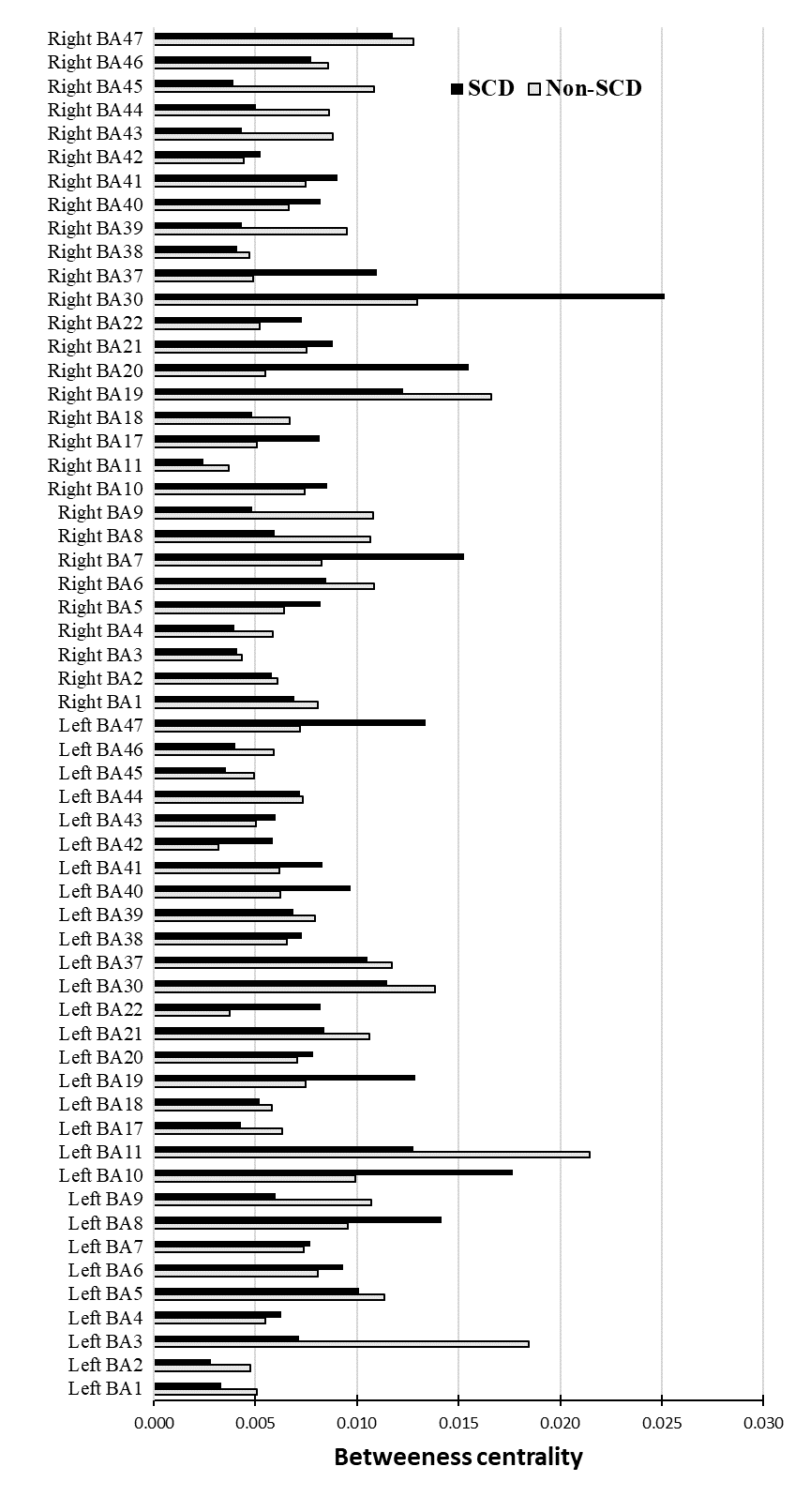

Supplement: Supplementary Figure 2 — Betweenness centrality (BC) scores of the 58 regions of interest (ROIs) in non-SCD and SCD group within the theta frequency band. The dotted line indicates the cut-off threshold. ROIs with BC scores above this cut-off were considered as hubs. [file Data_Sheet_2.docx]
